# Investigations on *Ananas erectifolius* fiber/sawdust hybrid epoxy composites for sustainable building applications

**DOI:** 10.1038/s41598-025-31748-x

**Published:** 2025-12-16

**Authors:** Palanivendhan Murugadoss, Kulmani Mehar, Yuvaraja Naik, Naveen Kumar Rajendran, Parin Kumar Patel, Kuldip Kumar Sahu, Deepali Dash, Kamakshi Priya Kumar

**Affiliations:** 1 Center for Automotive Materials, Department of Automobile Engineering, College of Engineering and Technology, SRM Institute of Science and Technology, Kattankulathur, Kanchipuram, 603203 Tamil Nadu India; 2 Department of Mechanical and Industrial Engineering, Manipal Institute of Technology, Manipal Academy of Higher Education, Karnataka, Manipal India; 3 Department of Mechanical Engineering, Presidency University, Bengaluru, Karnataka India; 4 Department of Aerospace Engineering, Faculty of Engineering and Technology, JAIN (Deemed-to-be University), Bangalore, Karnataka India; 5 Department of Chemical Engineering, Faculty of Engineering and Technology, Parul Institute of Technology, Parul University, Vadodara, Gujarat India; 6 Department of Mechanical Engineering, ARKA JAIN University, Jharkhand, India; 7 Department of Crop Physiology, Institute of Agricultural Sciences, Siksha ’O’ Anusandhan (Deemed to be University), Bhubaneswar, Odisha India; 8 Department of Physics, Saveetha School of Engineering, SIMATS, Saveetha University, Chennai, Tamil Nadu India

**Keywords:** Materials, Sustainable development, Natural fibers, Building materials, Material stability, Engineering, Materials science

## Abstract

This study investigates the mechanical, thermal, morphological, antibacterial, and water absorption characteristics of *Ananas erectifolius* fiber (AEF) reinforced epoxy composites filled with sawdust particulates, aiming to develop sustainable alternatives to conventional construction materials. Composites were fabricated using the hand layup method followed by compression molding, incorporating sawdust filler contents ranging from 0 to 24 g while keeping the fiber mass constant at 300 g. The composite containing 18 g of sawdust (Sample C3) demonstrated superior overall performance, achieving a tensile strength of 51.09 MPa, flexural strength of 54.98 MPa, impact strength of 14.98 kJ/m², and a Shore D hardness of 51. SEM analysis confirmed strong fiber–matrix interfacial bonding and uniform filler dispersion in the optimized formulation. Thermal assessments showed that Sample C3 exhibited the lowest thermal conductivity (0.72 W/mK), a reduced coefficient of linear thermal expansion (62.1 × 10⁻⁶/°C), and the highest heat deflection temperature (123 °C). TGA revealed enhanced thermal stability, with ~ 18% residual mass at 600 °C. Antibacterial testing against *E. coli* produced a 26 mm inhibition zone at 100 µg concentration. The water absorption rate remained low at 5.99%, indicating good dimensional stability. The novelty of this work lies in the Integrated valorization of two underutilized bio-wastes *Ananas erectifolius* fiber and sawdust to engineer a high-performance, eco-friendly hybrid composite tailored for sustainable building applications.

## Introduction

Traditional materials used for home interiors reflect a blend of natural aesthetics, durability, and cultural heritage. Wood remains a cornerstone, favoured for flooring, ceiling beams, and furniture due to its warmth and timeless appeal. Stone, such as marble and granite, is commonly used in flooring, countertops, and wall accents, offering both elegance and resilience. Clay and terracotta tiles are popular for their rustic charm and excellent thermal properties, often seen in traditional kitchens and courtyards^1^. Textiles like cotton, wool, and silk are widely used in curtains, upholstery, and rugs, adding softness and regional character^2^. One of the primary concerns is their susceptibility to moisture, which can lead to warping, swelling, or rotting over time, especially in humid environments. Wood is also vulnerable to termite and insect infestations, requiring regular treatment and maintenance^3^. Also, certain types of hardwoods used in traditional interiors may be sourced unsustainably, contributing to deforestation and environmental degradation. These limitations make it essential to balance traditional charm with modern treatments or alternatives to enhance performance and longevity^4^.

Natural fiber composites offer a sustainable and efficient alternative to traditional wood materials for home interiors, addressing many of their inherent drawbacks. By combining natural fibers such as jute, flax, hemp, or coir with biodegradable or synthetic resins, these composites provide enhanced resistance to moisture, reducing the risks of warping, swelling, and rot^5^. Unlike untreated wood, natural fiber composites are less susceptible to termite and insect damage, and they typically require minimal maintenance. Additionally, they are lightweight, durable, and can be engineered for specific mechanical and thermal properties, making them suitable for a wide range of interior applications such as panels, furniture, wall cladding, and decorative elements^6^. From an environmental perspective, natural fiber composites are often derived from renewable resources and can be more eco-friendly than hardwoods, helping to reduce deforestation and carbon emissions. Their versatility, cost-effectiveness, and aesthetic adaptability make them a promising solution for modern interior design with traditional appeal^7^. Natural fiber composites are increasingly being adopted as alternative materials to traditional wood in home interiors, with varying fiber content tailored to specific applications. These composites contain 30–60% natural fibers by weight, depending on the type of fiber and the matrix used^8^. For instance, jute- or flax-reinforced composites with 40–50% fiber content are commonly used in interior wall panels and partition boards due to their good strength-to-weight ratio and thermal insulation properties^9^. In furniture components and cabinetry, composites with 30–40% hemp or kenaf fibers are used to ensure dimensional stability and resistance to moisture^10^. Coir fiber composites with up to 60% fiber loading are employed in acoustic panels and ceiling tiles for their excellent sound absorption and lightweight characteristics^11^. These formulations not only enhance mechanical and environmental performance but also allow customization in texture and finish, closely mimicking the appearance of traditional wood while offering improved durability and sustainability^12^. Ananas *erectifolius* fiber and sawdust particles possess distinct physical properties that make them suitable for composite applications in home interiors. The fibers of *Ananas erectifolius*, a member of the pineapple family, typically exhibit an average length ranging from 30 to 120 mm and a diameter between 80 and 150 μm, depending on the extraction method and plant maturity^13^. These fibers are lightweight with a bulk density of approximately 1.20–1.40 g/cm³ and possess a relatively high cellulose content, contributing to their strength and stiffness. In contrast, sawdust particles, a common wood by-product, have a much smaller particle size, generally ranging from 100 to 1000 μm, depending on the mesh size used in processing^14^. Sawdust density varies based on wood species but typically falls within 0.25–0.65 g/cm³. The higher aspect ratio of *Ananas erectifolius* fibers compared to sawdust enhances their reinforcing potential in composite matrices, while the fine particle size of sawdust aids in filling voids and improving surface finish. These complementary properties enable their effective use in hybrid natural fiber composites for structural and decorative interior elements^15^.

Despite the increasing interest in natural fiber-reinforced polymer composites, limited research has addressed the synergistic effects of combining long, high-strength natural fibers with fine lignocellulosic fillers to enhance both structural and functional performance for construction applications. Most existing studies focus either on fiber or particulate reinforcement independently, neglecting the potential benefits of hybrid reinforcement systems. To bridge this gap, the present study introduces a novel composite system comprising *Ananas erectifolius* fiber (AEF) and sawdust particulates embedded in an epoxy matrix. The novelty of this work lies in its hybrid approach, which strategically integrates the superior tensile properties and high aspect ratio of AEF with the void-filling, stiffness-enhancing, and thermal insulation contributions of finely milled sawdust. This dual reinforcement mechanism is anticipated to significantly enhance the composite’s mechanical strength, thermal resistance, dimensional stability, and antimicrobial properties. The main objective of this research is to systematically investigate the effect of varying sawdust filler content on the mechanical, thermal, morphological, antibacterial, and water absorption characteristics of the AEF-reinforced epoxy composites, with the goal of identifying the optimal formulation for sustainable, durable, and eco-efficient applications in construction and interior design.

## Materials and experimental process

The materials used in this study include *Ananas erectifolius* fibers, sawdust particulates, and epoxy resin with corresponding hardener. Mature *A. erectifolius* leaves were harvested locally from the Tirunelveli district of Tamil Nadu, India, and fibers were extracted using a water retting process followed by sun drying. Commercial-grade sawdust, primarily from teak and neem wood, was procured from Sri Vignesh Timber Depot, Tirunelveli. The epoxy resin (LY 556) and hardener (HY 951) were purchased from Araldite^®^ authorized distributor M/s. Southern Polymers, Chennai. All materials were used as received without further chemical treatment to maintain eco-friendly processing.

### Fabrication process of AEF composite

The fabrication of chopped *Ananas erectifolius* fiber-reinforced epoxy composites with sawdust filler was carried out through a hand layup technique, followed by compression molding and thermal curing to ensure a uniform and mechanically robust laminate. This process begin with, *Ananas erectifolius* fibers were manually cleaned, cut to a standardized length, and oven-dried at 60 °C for 24 h to remove residual moisture, which is crucial for achieving strong fiber-matrix adhesion. Meanwhile, sawdust particles were sieved to ensure consistent particle size distribution and oven-dried to prevent moisture interference during mixing. The epoxy resin and sawdust filler were prepared in varying proportions as per the specified composition (C0 to C4), where the sawdust content ranged from 0 to 24 g and the epoxy content decreased correspondingly from 300 g to 276 g, maintaining a constant fiber mass of 300 g. The epoxy resin was first blended with sawdust filler using a mechanical stirrer at a speed of 600 rpm for 10 min to achieve a homogeneous dispersion of filler particles within the resin matrix. Following this, the curing agent (hardener) was added at a ratio of 10:1 (epoxy to hardener) and the mixture was further stirred at 400 rpm for an additional 5 min to activate the cross-linking mechanism without entrapping excess air bubbles^16^. The dried *Ananas erectifolius* fibers were then placed in a clean mold in an aligned and evenly distributed manner.

**Fig. 1:**
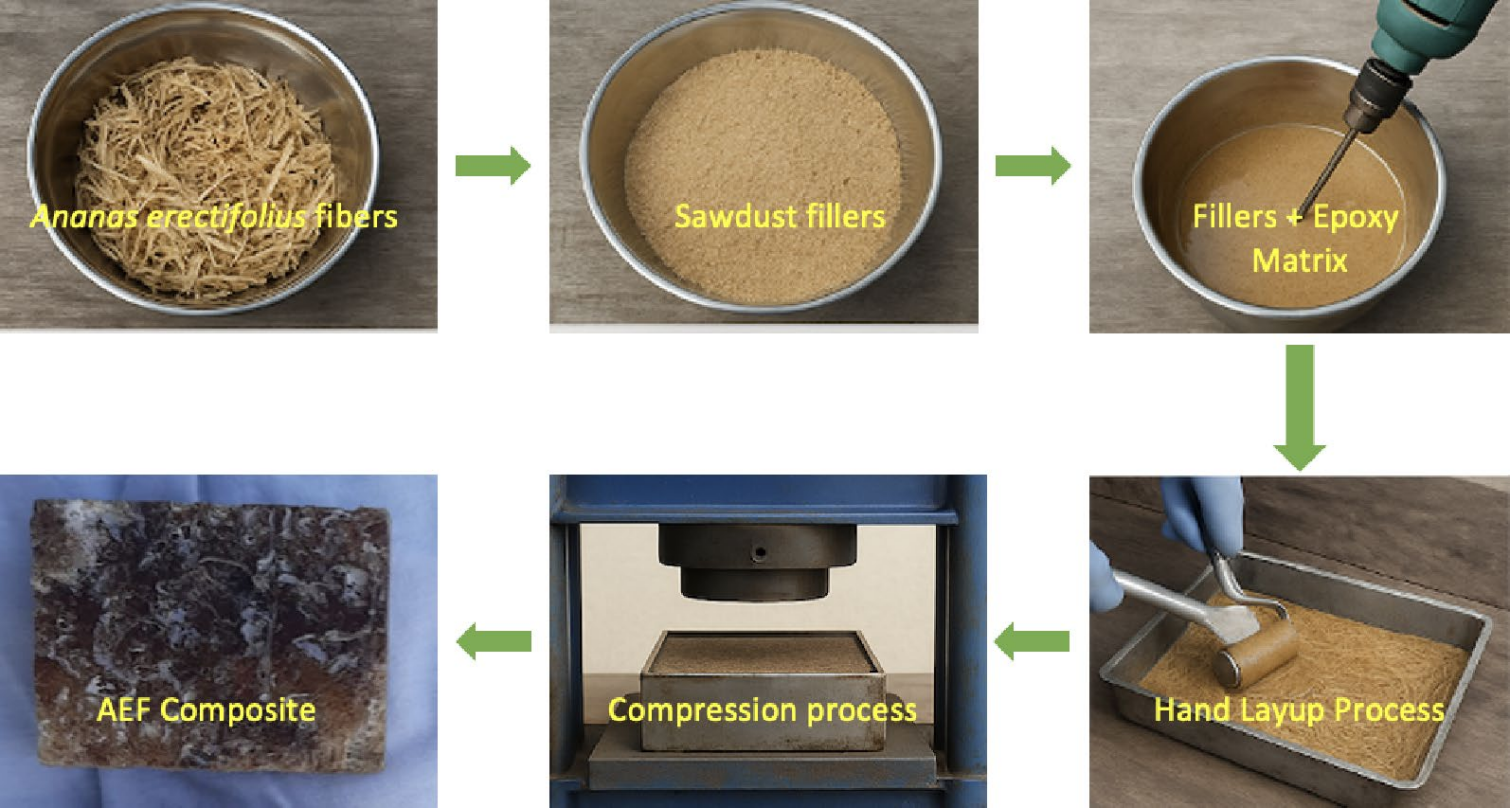
The fabrication process of AEF composite.

The prepared epoxy–sawdust mixture was gradually poured over the fiber bed in layers, with each layer being hand-rolled using a stainless-steel roller to eliminate trapped air and ensure thorough impregnation of the fibers. This step is vital for improving the wet-out and interfacial bonding between the hydrophilic natural fibers and the hydrophobic matrix. After complete layup, the mold was subjected to compression using a hydraulic press at a pressure of 12 MPa for 30 min to achieve consolidation and uniform thickness across the laminate. The compressed composites were allowed to cure at room temperature for 24 h, followed by a post-curing cycle in a hot air oven at 70 °C for 3 h to enhance the matrix cross-link density and improve thermal and mechanical stability^17^. Once fully cured, the composite panels were demoulded and trimmed to required dimensions for mechanical, thermal, and morphological testing. This multi-step fabrication protocol ensured consistent fiber alignment, uniform filler dispersion, reduced void content, and strong interfacial bonding resulting in bio-composites suitable for eco-friendly interior applications, offering improved strength, dimensional stability, and sustainability over traditional wood materials. Figure 1 shows the fabrication process of AEF composite. The weight ratio of AEF composite was given in the Table 1.

**Table 1:** The weight ratio of AEF composite.

Sample Code	AEF Fiber (g)	Sawdust (g)	Epoxy (g)	Total Mass (g)	Fiber (wt%)	Sawdust (wt%)	Epoxy (wt%)
C0	300	0	300	600	50.0%	0%	50.0%
C1	300	6	294	600	50.0%	1.0%	49.0%
C2	300	12	288	600	50.0%	2.0%	48.0%
C3	300	18	282	600	50.0%	3.0%	47.0%
C4	300	24	276	600	50.0%	4.0%	46.0%

### Testing process of *Ananas erectifolius* fiber

The experimental characterization of *Ananas erectifolius* fiber (AEF) composites filled with sawdust and embedded in an epoxy matrix was meticulously conducted to evaluate their mechanical, thermal, morphological, and antibacterial properties. These evaluations followed standardized testing protocols to assess the performance of the developed composites and their suitability as alternatives to conventional interior-grade wood materials. All tests were conducted in triplicate, and results are expressed as mean ± SD. Statistical significance was analyzed using ANOVA (*p* < 0.05).

#### Tensile testing (ASTM D3039)

Tensile properties, including tensile strength, tensile modulus, and elongation at break, were determined using a dual-column universal testing machine (Instron 3369, USA) with a 50 kN load cell. Specimens were prepared in accordance with ASTM D3039, having dimensions of 250 mm × 25 mm × 4 mm. Before testing, the samples were conditioned for 48 h at a controlled environment of 23 ± 2 °C and 50 ± 5% relative humidity. The test was carried out at a constant crosshead speed of 2 mm/min. Load and extension were continuously recorded, and stress-strain curves were generated to extract mechanical parameters. The tensile strength (σₜ) and tensile modulus (Eₜ) were calculated using: σt = Pmax/A, and Et = Δσ/Δε. The results provided the composite’s ability to withstand tensile loads and assess the reinforcing effect of sawdust filler^18^.

#### Flexural testing (ASTM D790)

Flexural performance was assessed using the same UTM setup in a three-point bending configuration. According to ASTM D790 standards, specimens of size 127 mm × 12.7 mm × 4 mm were used with a span-to-depth ratio of 16:1. The crosshead speed was set to 2 mm/min, and deflection was measured at the mid-span. The flexural strength and modulus were calculated using the load-deflection data. This test evaluated the stiffness and load-bearing ability of the composites under bending stress, which is critical for furniture and panel applications^19^.

#### Impact testing (ASTM D6110)

Impact resistance was measured using a Charpy impact tester (Tinius Olsen IT504, USA). The notched specimens were prepared as per ASTM D6110 with dimensions 80 mm × 10 mm × 4 mm and a V-notch depth of 2 mm. Each sample was tested under a single impact, and the absorbed energy was recorded in joules. This test provided essential data regarding the toughness and shock-absorbing capacity of the composites, which are important for ensuring durability in dynamic environments^20^.

#### Shore D hardness (ASTM D2240)

Surface hardness was evaluated using a Shore D durometer (TIME TH210, China) as per ASTM D2240. Flat specimens of 50 mm × 50 mm × 4 mm were tested. Five different points on each sample were measured, and the average was reported to account for surface heterogeneity. Shore D hardness reflects the material’s resistance to indentation and surface wear, important for flooring and wall panel applications^21^.

#### Scanning electron microscopy (SEM)

The microstructural morphology and interfacial bonding, fracture surfaces from tensile test specimens were examined using a scanning electron microscope (ZEISS EVO 18, Germany). Samples were gold sputter-coated using a Quorum Q150R ES coater for enhanced conductivity and observed at magnifications ranging from 100× to 2000×. SEM imaging provided insights into the dispersion of sawdust particles, fiber pull-out behavior, matrix cracking, and the quality of fiber-matrix adhesion, which influence the overall mechanical performance of the composite^22^.

#### Thermal conductivity (ASTM C177)

Thermal conductivity was evaluated using a guarded hot plate apparatus (Unitherm 2020, Holometrix, USA) following ASTM C177 standards. Disc-shaped specimens (100 mm diameter, 5 mm thick) were subjected to a temperature gradient from 10 °C to 50 °C. The steady-state method was employed to measure the rate of heat flow through the material. The principle of measurement is based on Fourier’s law of heat conduction, where heat flow at steady state is directly proportional to the temperature gradient. k= (Q⋅L)/(A⋅ΔT) where, *k* = thermal conductivity (W/mK), *Q* = heat flow rate through the specimen (W), *L* = thickness of specimen (m), *A* = cross-sectional area (m²), *ΔT* = temperature difference across faces (°C). This method quantifies the material’s ability to conduct heat and is essential for assessing insulation performance in building applications^23^.

#### Coefficient of linear thermal expansion (CLTE) (ASTM E831)

CLTE was measured using a thermomechanical analyser (TA Instruments Q400 TMA, USA). Samples of 10 mm × 5 mm × 4 mm were heated from 30 °C to 120 °C at a rate of 5 °C/min. The dimensional change with respect to temperature was recorded and used to compute the linear expansion coefficient. The dimensional change with temperature was recorded, and the coefficient of linear thermal expansion (α) was calculated using: α = ΔL/(L0⋅ΔT) where, *ΔL* = change in specimen length (mm), *L₀* = initial length (mm), *ΔT* = temperature change (°C) This data is crucial in determining the dimensional stability of the composite under thermal cycling^24^.

#### Heat Deflection temperature (HDT) (ASTM D648)

HDT was determined using a Ceast HDT/Vicat tester (Model: Ceast HDT 6, Italy) based on ASTM D648. Specimens (127 mm × 13 mm × 3 mm) were tested under a constant bending stress of 1.82 MPa while the temperature was increased at a rate of 2 °C/min. The temperature at which the sample deformed by 0.25 mm was recorded as HDT. This test evaluated the material’s capacity to retain its form under elevated temperatures, which is essential for structural applications near heat sources^25^.

#### Thermogravimetric analysis (TGA) (ASTM E1131)

Thermal degradation behavior was studied using a TGA analyser (Mettler Toledo TGA/DSC 1, Switzerland) under a nitrogen atmosphere to prevent oxidative degradation. Around 10 mg of the composite sample was heated from room temperature to 600 °C at a rate of 10 °C/min. The principle of TGA is based on monitoring the change in sample mass as a function of temperature or time. The mass loss (Δm) corresponding to thermal decomposition is quantified as: % Weight Loss = ((m_0_ – m_T_)/m_0_) x 100, where, m₀ = initial sample mass (mg), m_T_ = remaining mass at temperature T (mg). The TGA curve provided the onset degradation temperature, peak decomposition temperature, and residual char content. This helped in evaluating the thermal stability and suitability of the composite under thermal load^26^.

#### Water absorption test

The water absorption behavior of Ananas erectifolius fiber-reinforced epoxy composites containing sawdust filler was evaluated to assess their suitability for moisture-prone interior applications. The test was performed according to ASTM D570, which specifies the procedure for determining the water absorption capacity of polymer-based composites. Rectangular specimens with dimensions of 76.2 mm × 25.4 mm × 4 mm were cut and oven-dried at 60 °C for 24 h to eliminate any residual water absorption capacity. After cooling in a desiccator, the initial dry weight (W₀) of each sample was recorded using a high-precision digital balance (Shimadzu AUW220D).

The specimens were then fully immersed in distilled water at 23 ± 2 °C. At predetermined time period of 120 h, the samples were removed, gently wiped with blotting paper to remove surface water, and reweighed to obtain the wet weight (Wₜ). Water absorption percentage was calculated using the formula: (Wₜ − W₀)/W₀ × 100. This procedure was repeated for all composite formulations (C0 to C4), with three replicates per group to ensure accuracy. The water uptake behavior provided insight into the hydrophilic nature of the composites and the influence of sawdust content on their resistance in water absoprtion. This analysis is critical for determining the long-term dimensional stability and durability of the composites when used in environments exposed to humidity, such as kitchen cabinetry, bathroom partitions, or wall panels^27^.

#### Antibacterial Activity – Agar well diffusion method

The antibacterial efficacy of the composite samples was assessed using the agar well diffusion technique against *Escherichia coli* (Gram-negative). Mueller-Hinton agar plates were prepared, and 100 µL of bacterial suspension (10⁶ CFU/mL) was spread uniformly on the surface. Sterilized composite discs (10 mm diameter × 2 mm thick) were placed into wells (6 mm diameter) bored into the agar. The plates were incubated at 37 °C for 24 h, after which the inhibition zones were measured in millimetres using a digital caliper. The test indicated the antimicrobial potential of the composite, which is critical for hygienic interior applications, particularly in healthcare and kitchen environments^28^. This comprehensive testing regime ensured a multidimensional evaluation of AEF-based composites, revealing their mechanical integrity, thermal endurance, microstructural behavior, and antibacterial efficiency. The use of standardized methods and advanced equipment enabled precise and reproducible characterization, validating the composite’s performance for sustainable interior design and construction applications.

## Results and discussion

### Mechanical properties of AEF composite

**Fig. 2:**
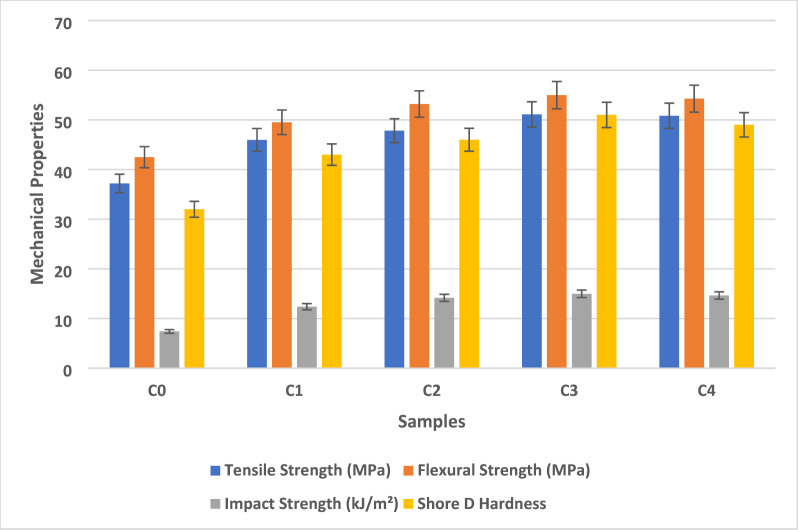
Mechanical properties of AEF composite.

Figure 2 shows the mechanical properties of AEF composite. The tensile strength behavior of AEF composites, revealed a distinct trend influenced by the filler loading and fiber–matrix interfacial dynamics. Among the five formulations (C0–C4), the composite sample C3 exhibited the highest tensile strength of 51.09 MPa, indicating a synergistic effect between the AEF fibers, sawdust particles, and the epoxy matrix. Compared to the control sample C0, which showed a tensile strength of 37.19 MPa, Sample C3 demonstrated a 37.4% enhancement, clearly reflecting the contribution of particulate reinforcement to mechanical performance. The improved tensile behavior with increasing sawdust content up to 18 g (C3) can be attributed to several reinforcing mechanisms. Firstly, the sawdust particles acted as effective micro-fillers, occupying the void spaces between the fibers and matrix was revealed in the SEM microstructure. This not only reduced internal porosity but also facilitated more uniform stress transfer under applied load. Secondly, the inclusion of sawdust moderately increased the viscosity of the resin, which proved beneficial for fiber wetting and impregnation, promoting stronger mechanical interlocking and better adhesion at the fiber–matrix interface.

Also, SEM analysis confirms that the irregular, micro-textured surfaces of the sawdust particles facilitate better mechanical interlocking with the epoxy matrix, contributing to improved interfacial bonding and enhanced mechanical properties. The lignocellulosic nature of both AEF and sawdust enabled hydrogen bonding with the polar epoxy matrix, further enhancing compatibility and stress transfer. However, at higher filler loading in Sample C4 (24 g of sawdust), the tensile strength slightly declined to 50.83 MPa. This marginal reduction is likely due to increased resin viscosity leading to poor resin flow, incomplete fiber impregnation, and potential particle agglomeration during the layup process. These microstructural defects act as stress concentrators and limit further strength improvements, suggesting a threshold beyond which filler content negatively affects composite performance. In comparison to conventional interior materials, such as medium-density fibreboard (MDF) and plywood, the tensile strength of the optimized composite (Sample C3) is notably superior. MDF exhibits tensile strength in the range of 20–25 MPa, while standard commercial plywood ranges between 30 and 40 MPa depending on the grade and orientation of plies^29^.

Therefore, the tensile strength of the AEF-sawdust epoxy composite (51.09 MPa) significantly exceeds that of these commonly used materials. This indicates that the developed composite not only meets but surpasses the mechanical requirements for interior structural applications such as wall panels, partition boards, cabinet components, and decorative elements, while also offering improved sustainability and reduced reliance on timber-based products. Moreover, unlike conventional wood-based panels, the AEF composite offers better dimensional stability, lower moisture sensitivity, and enhanced thermal resistance (as observed in supplementary thermal characterization), making it a more versatile and durable alternative^30^.

The incorporation of agro-waste materials such as *Ananas erectifolius* fibers and sawdust also aligns with circular economy principles and contributes to environmental sustainability by valorising agricultural residues and reducing deforestation pressure. Therefore, the tensile strength performance of the AEF/sawdust/epoxy composite, particularly in Sample C3, demonstrates a promising and superior alternative to traditional wood-based interior materials. The findings underscore the critical importance of optimizing filler loading and resin flow characteristics to maximize mechanical performance. Future research may explore functional surface treatments of fibers or hybridization with other bio-fillers to further enhance performance and broaden the application scope of such sustainable composite systems.

The flexural strength behavior of AEF composites filled with sawdust particles demonstrated a clear improvement in mechanical performance with increasing filler content up to an optimal level. Among the five composite formulations (C0–C4), Sample C3 exhibited the highest flexural strength at 54.98 MPa, indicating a well-balanced synergy between fiber reinforcement, particulate filler dispersion, and matrix characteristics. This represents a 29.4% increase compared to the control sample C0, which exhibited a flexural strength of 42.49 MPa. The progressive enhancement in strength observed in samples C1 (49.52 MPa) and C2 (53.19 MPa) supports the reinforcing effect of sawdust micro-fillers in improving stress transfer and structural integrity under bending loads. This improvement can be attributed to several key mechanisms.

The sawdust particles serve as effective micro-fillers that occupy the interstitial voids between the AEF fibers, thereby increasing the packing density of the composite and reducing internal defects such as microvoids or resin-rich regions. These filled regions enhance the distribution of bending stresses, minimizing the risk of localized failure. The rough surface texture and high aspect ratio of the lignocellulosic fillers contribute to mechanical interlocking and hydrogen bonding with the polar epoxy matrix, improving fiber–matrix adhesion and flexural stiffness^31^. The filler also acts to bridge microcracks during loading, thereby increasing energy absorption and delaying crack propagation. In terms of processing behavior, moderate addition of sawdust (up to 18 g in C3) results in a favorable increase in the viscosity of the resin, enhancing the wetting of fiber surfaces without compromising resin flow. This improved wetting promotes better interfacial adhesion, allowing more efficient stress transfer during flexural loading. However, beyond this optimal threshold, as observed in sample C4 (24 g sawdust), a slight reduction in flexural strength to 54.27 MPa was recorded. This decline can be attributed to excessive filler loading, which elevates the matrix viscosity to a level that restricts flow during hand layup, leading to incomplete fiber impregnation, void formation, and potential filler agglomeration^32^.

These defects reduce the cohesive strength of the composite and can serve as sites for stress concentration under bending. When compared to conventional interior construction materials, the flexural strength of the optimized AEF composite exceeds that of typical wood-based panels. Medium-density fibreboard (MDF) and oriented strand board (OSB) generally exhibit flexural strengths in the range of 20–40 MPa, while commercial plywood typically ranges between 30 and 50 MPa. Therefore, the AEF/sawdust/epoxy composite, with flexural strength values exceeding 54 MPa, presents a superior alternative in terms of mechanical performance.

Moreover, the composite offers additional benefits such as enhanced resistance in water absorption, thermal stability, and biodegradability characteristics that conventional wood-based materials often lack. Unlike MDF and plywood, which are susceptible to swelling and degradation in humid conditions, the AEF composite exhibits improved dimensional stability and environmental durability due to the hydrophobic nature of the epoxy matrix and the densified microstructure created by filler addition. Finally, the flexural strength results demonstrate that the AEF/sawdust/epoxy composite, particularly in the optimized C3 formulation, provides a mechanically robust and sustainable alternative to traditional interior materials. The superior bending performance, combined with favorable environmental and durability characteristics, supports its application in load-bearing interior components such as partition panels, cabinetry, and architectural elements. These findings emphasize the importance of optimizing filler content, resin viscosity, and fiber dispersion in the development of high-performance bio-based composites for structural interior use.

The impact strength of AEF composites, demonstrated significant enhancement in energy absorption capacity under sudden loading conditions. Among the five formulations (C0–C4), Sample C3 exhibited the highest impact strength of 14.98 kJ/m². This value represents with more positive influence when compared to the control sample C0, which recorded the lowest impact strength of 7.41 kJ/m². Intermediate increases were observed in Sample C1 (12.39 kJ/m²) and Sample C2 (14.17 kJ/m²), highlighting a consistent trend of improvement with incremental filler addition. The substantial improvement in impact strength, particularly in Sample C3, is primarily attributed to the optimized interaction between the AEF fibers, sawdust filler, and the epoxy matrix. The inclusion of 18 g of sawdust acted as an effective micro-reinforcement phase, bridging potential microcracks and dissipating impact energy more efficiently. These particles not only interrupted crack paths by acting as physical barriers but also enhanced the energy absorption capability by enabling micro-yielding zones around the filler–matrix interfaces^33^. Also, the interlocking effect due to the rough morphology of the sawdust particles promoted stronger mechanical anchoring, which played a vital role in resisting sudden fracture. From a processing perspective, the incorporation of filler content up to the optimal level (C3) resulted in moderate increases in matrix viscosity, which facilitated better wetting of the AEF fibers without severely compromising flowability. Improved fiber impregnation ensured uniform resin coverage, stronger fiber–matrix bonding, and better stress distribution under impact conditions. This enhanced interfacial adhesion helped transfer the applied dynamic load from the matrix to the reinforcing fibers and particles more effectively, minimizing localized failures. However, increasing the filler content to 24 g in Sample C4 resulted in a slight decline in impact strength to 14.64 kJ/m². This reduction, though marginal, is likely due to excessive viscosity impeding adequate fiber wetting and filler dispersion, leading to potential agglomeration and void formation that act as stress risers under impact. The comparatively low impact strength of the unfilled Sample C0 is due to the absence of filler-induced toughening mechanisms. In such systems, the crack propagates more readily along the fiber–matrix interface due to weak bonding and the lack of crack-arresting particles, resulting in brittle failure under impact. These findings indicate that Sample C3 achieves an optimal balance between filler content and matrix flow behavior, enhancing both energy absorption and crack resistance. The enhanced toughness of the optimized composite makes it suitable for structural applications where sudden or repeated mechanical shocks are expected, such as wall panels in high-traffic areas, cabinet doors, or other interior architectural components^34^. The results further emphasize the importance of controlling filler concentration and resin rheology in the fabrication of hybrid biocomposites to achieve improved toughness and reliability under impact loading.

The Shore D hardness evaluation of AEF composites incorporating sawdust filler revealed a significant enhancement in surface resistance to indentation with increasing filler content up to an optimal threshold. Among the five composite formulations (C0–C4), Sample C3 exhibited the highest Shore D hardness value of 51, marking a 59.4% increase compared to the unfilled control sample C0, which recorded a value of 32. Intermediate formulations showed a progressive rise in hardness, with Sample C1 and C2 reaching values of 43 and 46, respectively, indicating that sawdust functions as an effective micro-reinforcement phase in modifying surface hardness characteristics. The increase in Shore D hardness with the addition of sawdust can be attributed to multiple reinforcing mechanisms. Sawdust particles, being rigid and lignocellulosic in nature, enhance the microstructural stiffness of the composite by occupying resin-rich regions and reducing localized soft zones. These particles restrict polymer chain mobility at the surface and resist indentation forces more effectively than the epoxy matrix alone. The rough surface morphology and high aspect ratio of the sawdust also contribute to mechanical interlocking within the matrix, further increasing resistance to localized deformation.

From a processing standpoint, the incorporation of sawdust up to 18 g in Sample C3 results in a favorable increase in resin viscosity, which facilitates improved fiber wetting and more uniform filler dispersion. This contributes to a denser and more compact matrix structure with minimal voids, enhancing the composite’s ability to resist surface indentation. The improved interfacial bonding between the epoxy resin and both the AEF fibers and sawdust particles further enhances the hardness response by minimizing microstructural discontinuities at the surface. However, a slight reduction in Shore D hardness was observed in Sample C4, which recorded a value of 49. This decrease can be linked to the excessive filler loading (24 g), which likely increased the viscosity of the resin beyond the optimal processing range. High viscosity hinders adequate resin flow, leading to incomplete fiber impregnation and the formation of microvoids or agglomerates. These imperfections weaken surface consolidation and reduce resistance to localized stress, slightly compromising the hardness performance despite the higher filler content^35^.

The relatively low hardness value observed in the control sample C0 is indicative of an unreinforced matrix structure dominated by polymeric flexibility and lacking rigid particulate reinforcement. In this formulation, the stress from indentation is not efficiently distributed, resulting in higher surface deformation. The results clearly demonstrate that the addition of sawdust significantly enhances the surface hardness of AEF-reinforced epoxy composites, with Sample C3 presenting an optimal formulation. The improved Shore D hardness makes these composites highly suitable for interior applications requiring enhanced wear resistance and surface durability, such as furniture panels, shelving systems, and architectural trims. The findings also underscore the importance of carefully balancing filler content and matrix rheology to achieve desired surface properties in bio-based composite systems. The claim of mechanical interlocking and improved fiber–matrix adhesion is supported by the SEM observations of the fractured composite surfaces. The lignocellulosic sawdust particles exhibit a naturally rough and micro-textured surface, which allows the epoxy matrix to penetrate and anchor around the irregular features. In the optimized formulations, the SEM images show better particle embedding and fewer interfacial voids compared to the control sample, indicating enhanced physical interlocking at the interface. This microstructural evidence is consistent with the observed improvements in mechanical properties, confirming that the surface morphology of the sawdust fillers contributes to stronger interfacial bonding and increased stiffness.

### SEM microstructure of AEF composite

**Fig. 3:**
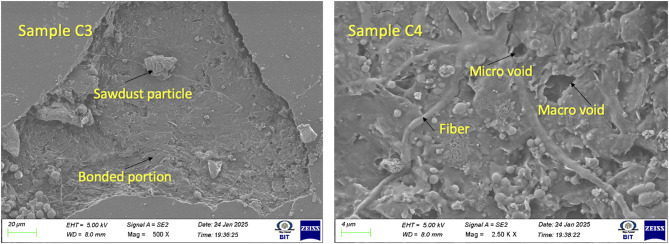
SEM microstructure of AEF composite.

The SEM microstructure of Sample C3 exhibits a highly cohesive and compact fracture surface, indicating an efficient load-bearing interface between the epoxy matrix and the sawdust filler. The micrograph reveals a continuous resin phase with uniformly embedded sawdust particles that appear well anchored within the matrix. The absence of clean pull-out regions and the presence of matrix residues adhered to the filler surfaces confirm strong adhesive bonding and effective stress transfer. The fracture surface is characterized by tortuous crack paths, micro-crack deflection zones, and roughened fracture planes—all of which are indicative of energy-dissipating mechanisms. This morphology suggests that during tensile loading, the crack propagation was repeatedly hindered and redirected by well-dispersed fillers, resulting in a semi-ductile fracture response. These microstructural features align with the superior tensile strength of C3, confirming that 18 g of sawdust provides an optimal reinforcement architecture in the AEF/epoxy system. In contrast, the SEM image of Sample C4 reveals a more heterogeneous microstructure with clear signs of microstructural deterioration due to excessive filler loading. Localized agglomerates of sawdust are visible, forming rigid clusters that disrupt the uniformity of the matrix. These agglomerated regions create stress concentrations where cracks can easily initiate. The presence of micro-voids, resin-starved patches, and partially detached filler particles underscores a decline in interfacial bonding quality^36^. Unlike C3, the crack path in C4 appears less tortuous, with several straight and rapidly propagating fracture zones indicative of a more brittle failure mechanism. The reduced connectivity between the resin and filler phases in C4 limits the ability of the composite to effectively arrest or deflect cracks, leading to slightly lower tensile strength compared to C3. Figure 3 shows the SEM microstructure of AEF composite.

Another notable distinction in Sample C3 is the presence of fibrillated fiber bundles that remain partially embedded in the matrix post-failure. This implies that fiber rupture, rather than interfacial debonding, contributed to the overall failure mechanism an indication of effective load transfer and improved tensile performance. The sawdust filler likely enhanced stress concentration distribution by reinforcing matrix-dominated zones, reducing the mismatch in mechanical properties at the fiber–matrix interface^37^. The overall microstructural compactness and continuity observed in Sample C3 are directly correlated with its superior tensile strength and toughness, as confirmed by mechanical testing. These comparative SEM analyses conclusively demonstrate that the addition of sawdust filler significantly improves the microstructural integrity of AEF composites by promoting stronger fiber–matrix interaction, minimizing void formation, and enabling more uniform stress dissipation under load. The optimized composition in Sample C3 exemplifies the beneficial effect of hybrid reinforcement in natural fiber composites, where particulate and fibrous phases act synergistically to improve fracture resistance and structural reliability. This evidence reinforces the importance of tailoring filler content, fiber dispersion, and matrix rheology in bio-composite design to achieve high-performance, sustainable materials suitable for structural and load-bearing applications.

### Thermal properties of AEF composite

**Fig. 4:**
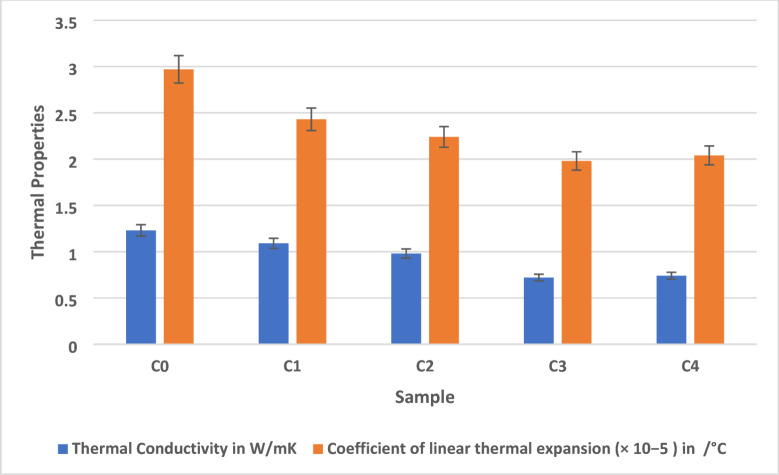
Thermal properties of AEF composite.

Figure 4 shows the thermal properties of AEF composite. The thermal conductivity analysis of AEF composites reveals a marked enhancement in thermal insulation performance with the inclusion of lignocellulosic filler^37^. Among the five formulations (C0–C4), Sample C3 exhibited the lowest thermal conductivity at 0.72 W/mK, representing a 41.5% reduction compared to the unfilled control sample C0, which registered the highest value at 1.23 W/mK. Samples C1 and C2 displayed intermediate values of 1.09 W/mK and 0.98 W/mK, respectively, showing a consistent downward trend with increasing filler content. Notably, Sample C4 showed a slight increase to 0.74 W/mK, indicating a potential plateau or reversal effect beyond the optimal filler loading. The presence of sawdust increases phonon scattering at the filler–matrix interfaces and introduces microvoids that disrupt continuous heat flow pathways^38^. This leads to a higher thermal resistance, especially in Sample C3, where the dispersion and volume fraction of the filler appear to reach an optimal balance, maximizing the disruption of conductive pathways within the matrix. Additionally, the inclusion of sawdust reduces the effective density of the composite, further enhancing its thermal insulating behavior. The marginal rise in thermal conductivity in Sample C4 suggests that beyond a certain threshold, excess filler may begin to agglomerate or reduce matrix-filler interfacial spacing, potentially forming localized conductive bridges that facilitate limited heat transfer.

This underscores the importance of optimizing filler concentration not only for mechanical strength but also for thermal insulation performance. From an application perspective, the superior thermal resistance of Sample C3 makes it particularly suitable for thermal insulation panels, false ceiling tiles, and partition walls in green buildings where heat retention or thermal barrier properties are desirable. Additionally, such composites can be effectively used in cabinet backboards, internal wall claddings, or insulated enclosures in residential and commercial interiors to reduce thermal bridging and improve energy efficiency. In packaging applications, the low thermal conductivity of C3 also renders it promising for insulated shipping containers or protective casing where temperature-sensitive goods are stored. Therefore, the incorporation of sawdust filler in AEF/epoxy composites significantly enhances their thermal insulation properties, with Sample C3 achieving the most favorable balance of filler dispersion, matrix continuity, and interfacial resistance. This result highlights the multifunctionality of bio-based composites, offering not only structural reinforcement but also targeted thermal regulation for sustainable and functional interior applications.

The CLTE is a vital thermal property that reflects a material’s dimensional stability when subjected to temperature fluctuations a critical consideration in composite materials used for structural and interior applications. In the current study, AEF composites filled with varying proportions of sawdust filler (C0–C4) were evaluated for their CLTE values. Among the samples, Sample C3 exhibited the lowest CLTE of 62.1 × 10⁻⁶/°C, representing a 48.2% reduction compared to the unfilled control sample C0, which recorded the highest CLTE at 119.8 × 10⁻⁶/°C. Intermediate reductions were observed in C1 (95.6 × 10⁻⁶/°C), C2 (78.4 × 10⁻⁶/°C), and C4 (65.7 × 10⁻⁶/°C), confirming that the inclusion of sawdust filler significantly improves the thermal dimensional stability of the composite up to an optimal threshold. This trend can be attributed to several interrelated mechanisms.

Firstly, sawdust, like AEF fiber, is a lignocellulosic material composed primarily of cellulose and lignin, which possess low thermal expansion coefficients compared to the epoxy matrix. As more of the polymeric phase is replaced by these rigid fillers, the overall CLTE of the composite decreases. Secondly, the inclusion of sawdust disrupts the continuity of the epoxy matrix and increases the number of interfaces within the material. These interfaces act as barriers to polymer chain mobility and reduce thermal strain, effectively restricting the expansion of the matrix during heating. In Sample C3, where the filler-to-matrix ratio is optimized, the sawdust is well-dispersed and efficiently bonded to the matrix, resulting in a uniform, constrained structure that minimizes expansion. The reduced thermal expansion in Sample C3 is also a direct consequence of improved interfacial adhesion between the matrix, sawdust particles, and AEF fibers. This strong interfacial bonding enhances mechanical interlocking and reduces free volume within the matrix, limiting thermal relaxation and chain movement^39^. In contrast, the higher CLTE observed in Sample C0 is due to the dominance of the epoxy phase, which expands more readily under thermal stress and lacks the constraining influence of filler particles. However, in Sample C4, despite a further increase in filler content (24 g), the CLTE slightly increases to 65.7 × 10⁻⁶/°C. This minor reversal is likely caused by filler agglomeration and reduced matrix wetting due to excessive viscosity, leading to the formation of interfacial defects or voids revealed in SEM microstructure.

These imperfections weaken the filler–matrix constraint effect, allowing localized thermal expansion and slightly reducing dimensional stability. This observation reinforces the importance of maintaining a balance between filler loading and processability to achieve optimal thermal performance. From an application standpoint, the low CLTE of Sample C3 makes it particularly advantageous for interior applications where thermal cycling is common, such as wall claddings, cabinet doors, ceiling panels, and decorative laminates. In these contexts, materials with low thermal expansion are essential to prevent warping, joint misalignment, and surface delamination, especially in humid or temperature-variable environments. Therefore, the significant reduction in CLTE observed in Sample C3 is a result of the combined effect on interaction between the natural fiber, sawdust filler, and epoxy matrix. This interaction restricts polymer chain mobility and reduces thermal strain through enhanced filler dispersion, interfacial adhesion, and microstructural integrity. The results clearly demonstrate that optimized hybrid natural fiber composites can be engineered not only for mechanical strength but also for superior thermal stability, supporting their use as eco-friendly alternatives in precision interior applications.

**Fig. 5:**
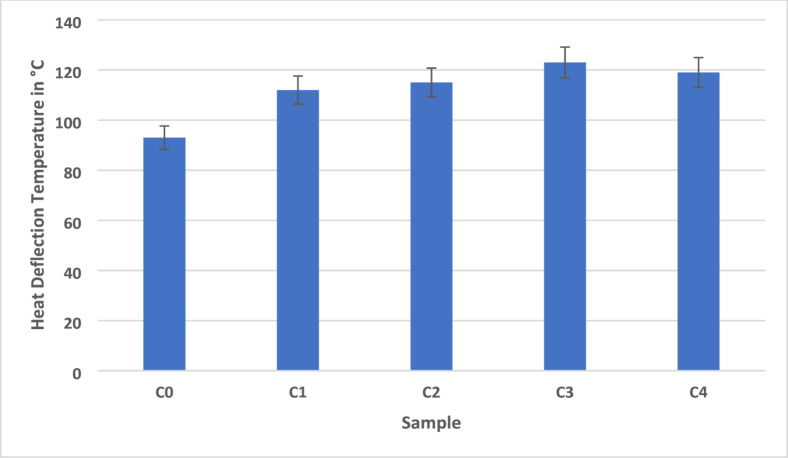
Short term heat resistance of AEF composite.

The HDT is a fundamental thermal property that defines the temperature at which a polymer composite begins to deform under a standardized load, making it a key indicator of a material’s ability to maintain dimensional stability under thermal stress. In this study, AEF composites were evaluated for their HDT characteristics was shown in the Fig. 5. Among the five composite formulations (C0–C4), Sample C3 exhibited the highest HDT value of 123 °C, demonstrating a notable improvement compared to the unfilled control, Sample C0, which recorded a lower HDT of 93 °C. The intermediate formulations showed a gradual enhancement with HDT values of 112 °C for Sample C1, 115 °C for Sample C2, and 119 °C for Sample C4, indicating that the inclusion of sawdust filler significantly improves thermal resistance. The increase in HDT can be attributed to the enhanced rigidity imparted by the lignocellulosic sawdust particles and natural fibers, which reduce the mobility of the epoxy matrix under thermal loading.

The thermally stable nature of the sawdust, rich in cellulose and lignin, introduces a reinforcing phase that absorbs and redistributes heat while restricting polymer chain movement. This effect is most prominent in Sample C3, where the optimal balance between fiber, filler, and matrix results in a dense microstructure with improved interfacial bonding, thereby delaying the onset of matrix softening and deformation. The slight decrease in HDT observed in Sample C4, despite a higher filler content, may be attributed to the onset of filler agglomeration or insufficient matrix wetting.

Excess filler can lead to poor dispersion, voids, or weak fiber–matrix interfacial regions, which reduce the composite’s ability to resist thermal deflection under load^40^. This emphasizes the importance of optimizing filler content to ensure uniform distribution and efficient stress transfer across the composite structure. Sample C3 demonstrates improved thermal performance relative to the other formulations, as reflected by its lower thermal conductivity and higher heat-deflection temperature; however, this should be interpreted as enhanced thermal resistance within the context of this study rather than definitive thermal stability for all high-temperature applications. The ability of this composite to maintain mechanical integrity and dimensional consistency under heat enhances its durability and performance compared to conventional wood-based materials or unreinforced epoxy systems. This improvement is attributed to increased matrix rigidity, enhanced filler–matrix interaction, and optimal filler dispersion, making such composites highly promising for thermally demanding and sustainable interior applications.

### Thermal stability of AEF composite

**Fig. 6:**
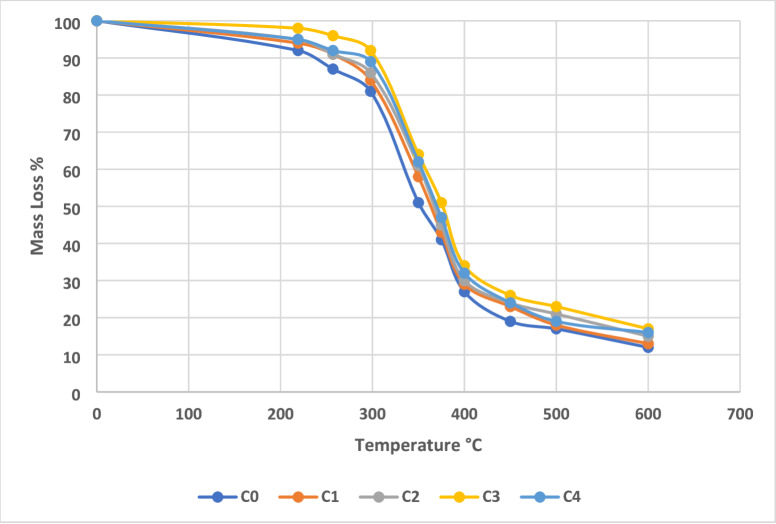
TG curve of AEF composite.

The thermogravimetric (TG) and derivative thermogravimetric (DTG) analyses reveal significant improvements in the thermal degradation resistance of AEF-based composites with the incorporation of sawdust filler. The unfilled control composite (Sample C0) exhibited a total mass loss of approximately 82.8% at 600 °C, while Sample C3, containing 18 g of sawdust filler, showed a reduced mass loss of 77.2%, indicating enhanced thermal stability. The residual char content at 600 °C increased from 17.2% in C0 to 22.8% in C3, demonstrating improved thermal insulation and higher carbonaceous residue generation due to the lignin-rich sawdust and fibrous reinforcement. This increase in char yield is crucial for applications requiring flame retardancy and structural integrity under thermal load^41^. Figure 6 shows the TG curve of AEF composite.

**Fig. 7:**
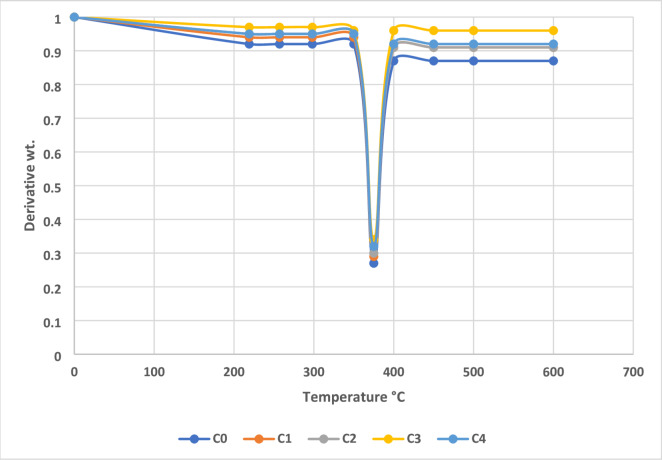
DTG curve of AEF composite.

In the DTG analysis, Sample C0 displayed a peak decomposition temperature around 368 °C, while Sample C3 exhibited a broader, shifted peak near 374 °C, suggesting a more controlled and gradual degradation process. The broader DTG peak in C3 is attributed to improved dispersion and interaction of the filler within the matrix, which restricts thermal diffusion and volatile release. This effect is amplified by the synergistic interaction between *Ananas erectifolius* fibers and sawdust particles, which together form a compact, thermally insulating microstructure that delays the onset of degradation. These enhancements are driven by mechanisms such as improved filler–matrix adhesion, reduced polymer chain mobility, and better thermal shielding provided by the lignocellulosic content. The thermally stable filler particles absorb and dissipate heat more effectively than the matrix alone, while the increased char acts as a barrier to heat and mass transfer during decomposition^42^. In contrast, excessive filler content, as in Sample C4, leads to agglomeration and possible microvoids, which reduce the uniformity and thermal resistance of the composite. Figure 7 shows the DTG curve of AEF composite.

When compared to conventional interior materials such as medium-density fibreboard (MDF) and particleboard, which typically begin to degrade significantly above 280–300 °C with lower char residue (~ 10–15%), the AEF-based composite particularly Sample C3 offers superior thermal performance. The higher thermal degradation temperature and increased residual mass make it a better alternative for applications requiring resistance to prolonged or elevated thermal exposure, such as partition walls near appliances, heat-exposed cabinetry, and structural panels in hot environments^43^. The TG and DTG results confirm that Sample C3 demonstrates the best balance between filler content and thermal stability. The improved degradation resistance over conventional wood-based products highlights the potential of AEF/sawdust/epoxy composites as a sustainable, thermally durable substitute for interior construction and furnishing materials.

### Antibacterial activity of AEF composite

The antibacterial evaluation of AEF composites against *Escherichia coli* revealed a distinct concentration-dependent efficacy, with inhibition zones measuring 19 mm for the 50 µg sample and 26 mm for the 100 µg sample. This represents a progressive improvement in antibacterial performance as the concentration of the active composite material increases. The 100 µg AEF composite achieved an inhibition zone nearly equivalent to that of the reference antibiotic, Streptomycin (10 µg), which exhibited a zone of 27 mm. This narrow difference highlights the potential of AEF composites to function as effective natural antibacterial materials in practical applications.

**Fig. 8:**
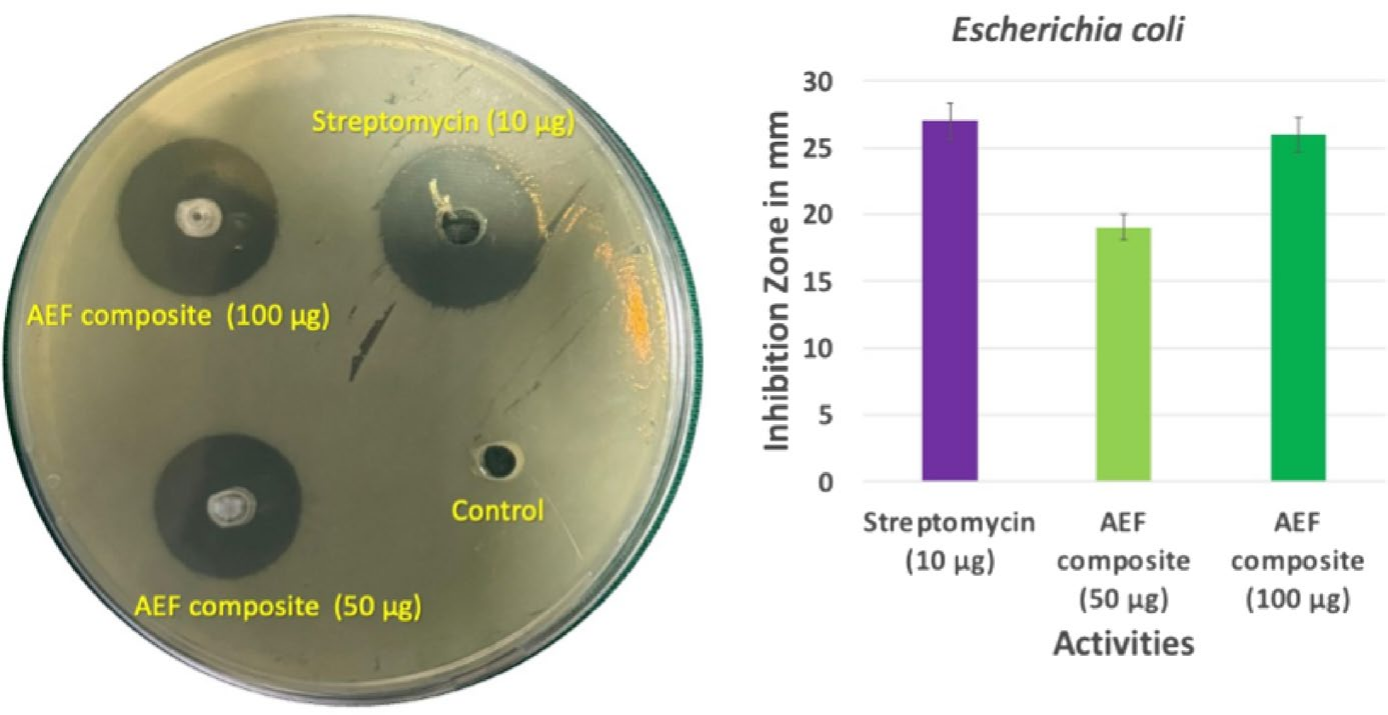
Antibacterial activities of AEF composite.

The enhanced inhibition observed at higher concentrations is primarily attributed to the presence of intrinsic antimicrobial compounds within the fiber structure, such as lignin-derived phenolics, flavonoids, and tannins^44^. These compounds interfere with bacterial cell integrity by inducing oxidative stress, disrupting membrane permeability, and impairing essential metabolic pathways. In addition, the porous microstructure of the composite facilitates better diffusion of these bioactive components into the agar medium, resulting in a broader inhibition zone. The interaction is further supported by the composite’s hydrophilic character, which enhances water absorption and promotes the localized release of antibacterial agents. The role of the epoxy matrix should also be considered. It acts not only as a binding phase but as a medium that may enable slow and sustained leaching of the active compounds over time. This controlled release mechanism ensures that bacterial growth is suppressed consistently rather than through a rapid, short-lived burst of activity. Moreover, the increased surface roughness due to the embedded fiber and sawdust content potentially augments physical disruption of bacterial colonies at the contact interface, adding a mechanical component to the antimicrobial action. Figure 8 shows the antibacterial activities of AEF composite.

AEF composites show antibacterial activity because *Ananas erectifolius* fibers naturally contain bioactive compounds such as phenolics, flavonoids, tannins, and lignin-derived extractives, which are known to inhibit bacterial growth. Their presence can be indirectly supported by FTIR peaks associated with O–H, C–O, and aromatic lignin structures. Unlike plywood or MDF, which lack such intrinsic compounds^45^, AEF composites derive their antibacterial effect from these naturally occurring constituents. Those traditional materials often require chemical coatings or additives to achieve even minimal antibacterial activity, which can raise concerns about environmental toxicity and long-term health exposure. In contrast, AEF composites derive their antibacterial performance from natural constituents, offering a safer, eco-friendly alternative that combines structural function with hygienic performance. These results affirm the multifunctionality of AEF composites, particularly for indoor applications in health-sensitive or humid environments such as kitchen cabinets, wall linings, bathroom partitions, and hospital furniture. Their ability to suppress microbial proliferation without chemical additives enhances their suitability for green building initiatives and sustainable material innovation. The near-parity in performance with Streptomycin underscores the promise of fiber-based biocomposites as viable antimicrobial alternatives in non-pharmaceutical, material-based interventions.

### Water absorption capacity of AEF composite

**Fig. 9:**
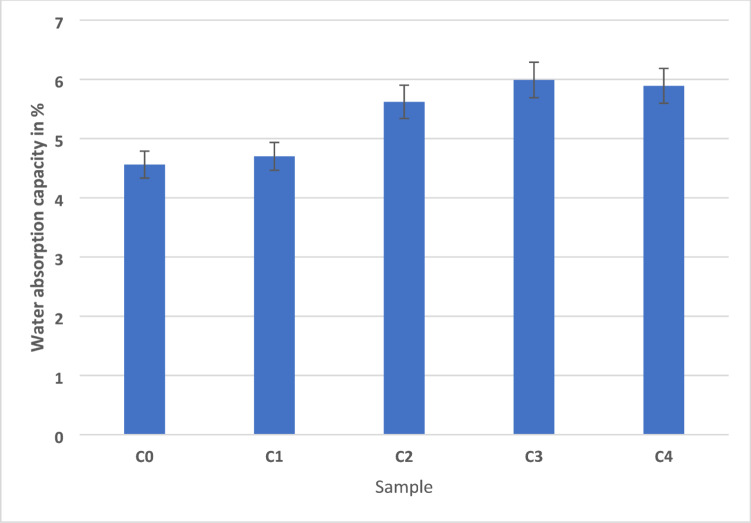
The water absorption capacity of AEF composite.

The water absorption capacity of AEF composites was shown in the Fig. 9, provides critical insights into their suitability for applications in moisture-prone interior environments. The measured values demonstrate a clear trend with respect to sawdust filler content, ranging from 4.56% in sample C0 to a peak of 5.99% in sample C3. Specifically, sample C0, which lacks any sawdust filler and contains only fiber and epoxy, exhibited the lowest absorption at 4.56%. This is primarily attributed to the dense epoxy network that effectively encapsulates the fiber content, limiting water ingress by reducing microvoids and surface porosity. Sample C1, with 6 g of sawdust, showed a slight increase to 4.70%, suggesting the initial introduction of filler begins to marginally disturb the homogeneity of the matrix but does not significantly compromise its barrier properties. A more pronounced increase is observed in samples C2, C3, and C4 with filler contents of 12 g, 18 g, and 24 g respectively. Sample C2 registered a water absorption of 5.62%, while samples C3 and C4 showed the highest values at 5.99% and 5.89%, respectively.

The peak in sample C3 corresponds to the optimal filler content where a balance is struck between filler dispersion and matrix encapsulation, resulting in increased surface roughness, microchannel formation, and enhanced capillary absorption^46^. These values reflect the inherent hydrophilicity of both sawdust and AEF fibers, which possess polar hydroxyl groups that readily interact with water molecules through hydrogen bonding. The increased filler concentration also disrupts the continuity of the epoxy matrix, generating more microvoids and interfacial gaps that serve as potential water diffusion pathways. Interestingly, the slight decline in water absorption in sample C4 compared to C3 may be explained by partial agglomeration of excess sawdust filler. This agglomeration likely reduces effective surface area and limits the number of active capillaries available for moisture diffusion, thereby marginally lowering water uptake. Furthermore, the increased packing density in C4 might reduce matrix discontinuities, thereby slightly improving resistance to water penetration despite higher filler content. In comparison to conventional interior-grade materials like MDF, plywood, and particleboard which absorb between 8% and 14% absorption under standard atmospheric conditions the AEF composites exhibit significantly improved water resistance.

This enhanced dimensional stability is advantageous for furniture, cabinetry, and panelling in kitchens, bathrooms, and humid indoor settings. Additionally, the incorporation of epoxy resin contributes to the hydrophobic nature of the composite surface, thereby delaying the onset of swelling, delamination, or microbial degradation associated with wood-based substrates. From a materials engineering standpoint, these results underscore the importance of optimizing filler content to control water absorption while maintaining structural integrity^47^. The moderate water uptake observed in AEF composites can be further reduced through surface treatments such as alkali modification, silane coupling agents, or water-repellent coatings, making them even more robust for high-moisture environments. Overall, the water absorption behavior of these composites confirms their competitive edge over traditional wood-based materials in terms of longevity, dimensional stability, and eco-efficiency in indoor applications.

## Conclusion

This study demonstrated the successful fabrication and evaluation of Ananas erectifolius fiber–reinforced epoxy composites filled with sawdust as a sustainable alternative to conventional wood-based materials. Among the formulations, Sample C3 (18 g sawdust) showed the most balanced overall performance, with notable improvements over the control in tensile, flexural, impact strength, and hardness, alongside reduced thermal conductivity, lower CLTE, and higher HDT. TGA confirmed higher residual char yield, indicating better thermal endurance, while antibacterial testing showed a 26 mm inhibition zone against *E. coli*, comparable to streptomycin. Water absorption increased only slightly, maintaining good dimensional stability. With its combined mechanical strength, thermal insulation capacity, microbial resistance, and environmental benefits, the optimized AEF–sawdust composite demonstrates strong potential for interior applications such as wall cladding, ceiling panels, cabinetry, and hygienic environments, positioning it as a sustainable and viable substitute for traditional wood-based and synthetic interior materials.

## Data Availability

The datasets used and/or analysed during the current study available from the corresponding author on reasonable request.
